# Rethinking Thiamine in the Emergency Department: Why a Hi-Phy-Vi-Based “Suspect and Treat” Approach Matters in an Aging Society

**DOI:** 10.7759/cureus.103003

**Published:** 2026-02-04

**Authors:** So Sakamoto

**Affiliations:** 1 Department of Emergency Medicine, Asahi General Hospital, Asahi, JPN

**Keywords:** diuretics, emergency department, frailty, gastrointestinal beriberi, lactate, thiamine deficiency, wernicke encephalopathy

## Abstract

Thiamine deficiency remains a clinically relevant yet frequently overlooked condition in modern emergency departments, particularly in aging societies such as Japan. Although traditionally regarded as a rare or historical disease, thiamine deficiency is not uncommon among emergency patients with high-risk backgrounds, including older adults, malnutrition, malignancy, chronic illness, diuretic use, gastrointestinal surgery, pregnancy-related vomiting, and social isolation. Despite its prevalence, diagnosis is often delayed or missed because of nonspecific presentations, reliance on incomplete classic triads, and dependence on laboratory confirmation that is neither rapid nor widely available in emergency settings. Importantly, thiamine deficiency represents a continuum of disease spanning early, nonspecific symptoms (including gastrointestinal complaints) to overt syndromes such as Wernicke encephalopathy (WE) and wet beriberi. While Wernicke encephalopathy and wet beriberi are well recognized, earlier and less familiar manifestations, such as gastrointestinal beriberi characterized by nausea, vomiting, anorexia, and abdominal discomfort, are easily misattributed to benign or self-limited conditions. Failure to recognize these early stages may allow progression to irreversible neurological or cardiac injury. In the emergency department, thiamine deficiency should therefore be approached as a diagnostic safety issue rather than a rare metabolic disorder. Clinical decision-making should prioritize history, physical examination, and vital signs (Hi-Phy-Vi) over delayed laboratory testing. Given the low cost and favorable safety profile of parenteral thiamine, a “suspect and treat” strategy based on clinical risk assessment is more appropriate than a “test and wait” approach. This editorial rethinks the role of thiamine in emergency care, emphasizing that early recognition and empiric treatment guided by bedside assessment can prevent diagnostic error and improve patient outcomes.

## Editorial

Thiamine (vitamin B1) is an essential water-soluble vitamin required for aerobic glucose metabolism and energy production, particularly in organs with high metabolic demand such as the brain and myocardium. Because total body stores are limited, approximately 30 mg, thiamine deficiency may develop within only two to three weeks of inadequate intake [1,2].

In contemporary emergency departments, especially in aging societies, patients frequently present with backgrounds that predispose them to thiamine depletion. These include older age, frailty, malignancy, chronic illness, malnutrition, gastrointestinal surgery, dialysis, diuretic use, pregnancy-related vomiting, alcohol use, and social isolation. Emergency department-based studies have demonstrated that thiamine deficiency remains common and underrecognized among hospitalized emergency patients, including vulnerable subgroups such as oncology patients [3,4]. In critically ill patients with septic shock, the reported prevalence is even higher [5]. Table 1 summarizes common high-risk backgrounds encountered in emergency department patients.

**Table 1:** High-Risk Backgrounds for Thiamine Deficiency in Emergency Department Patients

Category	High-risk background
Age/function	Older age, frailty, recent functional decline
Nutrition	Poor intake, anorexia, weight loss, malnutrition
Gastrointestinal	Prolonged vomiting, hyperemesis gravidarum, gastrointestinal surgery
Chronic disease	Malignancy, chronic inflammatory disease, heart failure
Renal/metabolic	Dialysis, chronic kidney disease
Medications	Loop or thiazide diuretics
Substance use	Alcohol use
Critical illness	Sepsis, septic shock
Social factors	Social isolation, limited access to food

Despite this, thiamine deficiency continues to be perceived as a historical or uncommon diagnosis. This mismatch between prevalence and clinical awareness creates a substantial risk for diagnostic error in emergency practice.

Wernicke encephalopathy (WE) is the most widely recognized manifestation of thiamine deficiency and is classically described by the triad of altered mental status, ocular motor abnormalities, and gait ataxia. However, this triad is present in only a minority of cases, and approximately 80% of patients are reportedly missed at initial presentation [6]. Delayed recognition may result in irreversible neurological injury, progression to Korsakoff syndrome, or death. Several misconceptions contribute to these missed diagnoses. WE is often incorrectly regarded as a condition limited to patients with alcohol dependence, despite the wide range of non-alcoholic risk backgrounds common in emergency departments [6]. Pathophysiologically, the shared denominator is impaired aerobic glucose metabolism leading to regional cerebral energy failure in susceptible brain networks, which can be precipitated by any cause of acute thiamine depletion or increased demand, not only alcohol dependence. Clinicians may also wait for the full triad to appear before considering the diagnosis, even though early intervention should be initiated based on partial or evolving findings. Furthermore, once an alternative diagnosis, such as stroke, infection, or heart failure, is identified, thiamine deficiency is frequently excluded, despite the common coexistence of multiple conditions in older or critically ill patients.

Thiamine deficiency should be understood as a continuum rather than a single disease entity. While WE and wet beriberi represent advanced stages, earlier manifestations are less specific and therefore more easily overlooked. Gastrointestinal beriberi is one such early manifestation, characterized by nausea, vomiting, anorexia, abdominal pain, and general malaise. This condition represents a forme fruste of thiamine deficiency that may precede overt neurological or cardiac involvement [7]. In emergency settings, these symptoms are often attributed to benign gastrointestinal illness, particularly in older adults with poor nutritional status, allowing progression to more severe disease.

Similarly, wet beriberi presents as high-output heart failure that may later transition to low-output failure [8]. In addition to high-output physiology, beriberi heart disease can present with prominent right-sided heart failure findings and acute pulmonary hypertension, which may improve rapidly after thiamine repletion [9]. In older adults and patients with heart failure, thiamine insufficiency has been increasingly reported, and diuretic use has been associated with lower thiamine levels and higher brain natriuretic peptide values, suggesting that empiric diuretic therapy may paradoxically worsen the underlying condition [2]. Pregnancy-related vomiting represents another high-risk scenario, as hyperemesis gravidarum is a well-established risk factor for thiamine deficiency and WE, with potentially catastrophic maternal and fetal outcomes if untreated [10,11].

Laboratory assessment of thiamine status, including whole blood thiamine levels, thiamine diphosphate concentration, or erythrocyte transketolase activity, has important limitations in emergency care. These tests are not widely available, often require external processing, and typically take several days to return results, limiting their utility for acute decision-making [12]. Furthermore, measured levels do not always reflect tissue-level deficiency or functional impairment. In addition, static circulating measurements may not capture dynamic cellular demand or transporter-mediated uptake/utilization during acute illness, reinforcing why bedside clinical assessment and early empiric treatment remain central.

Accordingly, emergency clinicians must rely on bedside assessment. A Hi-Phy-Vi-based approach emphasizes patient history, physical examination, and vital signs, integrating subtle neurological, gastrointestinal, and circulatory findings. When lactate is elevated, it should be interpreted as a marker of metabolic stress and impaired oxidative metabolism, not solely as a marker of hypoperfusion, and thiamine deficiency should remain on the differential in the appropriate clinical context [13]. The Caine criteria, which require two of four clinical features for the diagnosis of WE, align naturally with this framework and support early clinical suspicion [14]. A practical Hi-Phy-Vi-based bedside safety net for identifying patients at risk of thiamine deficiency in the emergency department is summarized in Table 2.

**Table 2:** Hi-Phy-Vi-Based Thiamine Safety Net in the Emergency Department

Domain	Clinical clue 1	Clinical clue 2	Clinical clue 3	Clinical clue 4	Clinical clue 5	Clinical clue 6	Clinical clue 7	Clinical clue 8	Clinical clue 9	Clinical clue 10	Clinical clue 11
History (Hi)	Reduced intake ≥2-3 weeks	Anorexia	Persistent nausea/vomiting	Hyperemesis gravidarum	Frailty	Malignancy	Chronic illness	Gastrointestinal surgery	Diuretic use	Alcohol use	Social isolation
Physical (Phy)	Altered mental status	Ocular abnormalities	Gait disturbance/ataxia	Peripheral neuropathy	Unexplained weakness		-	-	-	-	-
Vital signs and physiology (Vi)	Unexplained tachycardia	Lactate elevation	Diuretic-resistant heart failure	Evolving circulatory instability			-	-	-	-	-
Action	Initiate parenteral thiamine without waiting for laboratory confirmation when high-risk history and compatible bedside findings coexist										

Parenteral thiamine administration is inexpensive and generally safe. Although allergic reactions have been reported, serious adverse events are rare; many protocols administer thiamine as a slow intravenous infusion (e.g., over ~30 minutes) rather than a rapid push [15,16]. In contrast, the consequences of delayed or missed treatment, including irreversible neurological injury and death, are substantial. For patients with suspected WE or advanced deficiency, commonly used high-dose parenteral regimens include thiamine 500 mg IV three times daily for 2-3 days (and in some protocols 3-5 days), followed by 250 mg IV/IM once daily for a further 3-5 days or until clinical improvement plateaus [16,17]. For prophylaxis or early suspicion in high-risk patients with nonspecific symptoms, empiric supplementation such as 100-250 mg IV/IM once daily with transition to oral therapy when feasible is commonly used [16,17].

In aging societies, thiamine deficiency represents a common yet underappreciated threat to diagnostic safety in emergency care. By prioritizing bedside assessment over delayed laboratory confirmation, emergency clinicians can reframe thiamine not as a confirmatory therapy, but as a low-risk intervention that prevents high-stakes diagnostic failure.
